# Unleashing immuno-mass spectrometry superpowers to detect SARS-CoV-2

**DOI:** 10.1016/j.ebiom.2021.103480

**Published:** 2021-07-10

**Authors:** Jean Armengaud

**Affiliations:** Université Paris-Saclay, CEA, INRAE, Département Médicaments et Technologies pour la Santé (DMTS), SPI, 30200 Bagnols-sur-Cèze, France.

**Keywords:** Mass spec, Immuno-capture, SARS-CoV-2

## Abstract

In this article of EBioMedicine, Santosh Renuse and colleagues^1^ show the relevance of combining immunoaffinity capture with targeted mass spectrometry measurement to detect SARS-CoV-2 nucleocapsid proteins in nasopharyngeal swab samples. The COVID-19 pandemic has confirmed the need to improve the toolbox available to diagnose respiratory infections. Rapid, reliable, and highly specific detection is essential if we are to mount immediate preventive and therapeutic responses. This report stands out from previous studies as it implements immunocapture along with robust validation for a large cohort of subjects. The results presented show that mass spectrometry is definitively at a crossroads for large-scale clinical applications.

In this article of EBioMedicine, Santosh Renuse and colleagues [1] show the relevance of combining immunoaffinity capture with targeted mass spectrometry measurement to detect SARS-CoV-2 nucleocapsid proteins in nasopharyngeal swab samples. The COVID-19 pandemic has confirmed the need to improve the toolbox available to diagnose respiratory infections. Rapid, reliable, and highly specific detection is essential if we are to mount immediate preventive and therapeutic responses. This report stands out from previous studies as it implements immunocapture along with robust validation for a large cohort of subjects. The results presented show that mass spectrometry is definitively at a crossroads for large-scale clinical applications.

Molecular identification of specific regions of SARS-CoV-2 viral RNA by reverse transcription polymerase chain reaction (RT-PCR) has proven to be a valuable tool in COVID-19 diagnosis and for the epidemiological investigation of variants of concern. However, some issues related to the viral RNA target have been raised, such as the fact that some patients continue to produce a positive RT-PCR result for several weeks after initial infection despite the absence of evidence for viral replication [2]. Additionally, the RT-PCR method provides variable results for samples in which the RNA load is low due to stochastic effects during primer-target-matrix interactions.

In contrast, viral proteins produced in infected individuals are more closely tied to the infectious period, and thus, represent ideal targets for screening. Lateral flow immunoassays were developed to detect the SARS-CoV-2 spike protein, and are both fast and a valid alternative to mass testing. Unfortunately the method lacks sensitivity. In addition, because this approach requires specific antibody reagents, it is not compatible with an immediate response to the emergence and quick spread of a new pathogen of concern.

The method developed by Renuse and colleagues [1], relies on mass spectrometry, which is fast, sensitive, highly discriminative, and can simultaneously monitor a broad panel of targets. For over a decade now, protein masses have been profiled by whole-cell MALDI-TOF in many microbiological laboratories to instantly identify bacterial isolates grown on agar plates. Tandem mass spectrometry is even more discriminative and can be directly applied to complex samples without any need for pathogen amplification by culture [3]. In the near future, it could become the reference methodology for diagnosis in virology.

In the early months of the COVID-19 pandemic, tandem mass spectrometry showed its potential to become a credible alternative for diagnosis. The most relevant peptides from the SARS-CoV-2 capsid for use as tandem mass spectrometry markers were rapidly reported [4]. The list was further improved by taking into account the numerous SARS-CoV-2 genome sequences produced [5]. Several targeted proteomic approaches were then proposed to identify the main viral components present on nasopharyngeal swabs [6] and in gargle samples [7]. However, these proof-of-concept studies presented data from only few clinical samples. In their report, Renuse and colleagues [1] extend the sensitivity of the approach by introducing SARS-CoV-2 immunocapture and filtering the ions using a differential ion mobility device before mass measurement. They successfully stressed the methodology by testing it on samples with low viral load. After optimization, they performed the assay on a very respectable 176 nasopharyngeal swab specimens to estimate its sensitivity and specificity. The results of this cohort study showed outstanding performance – with a sensitivity of 98% and a specificity of 100% relative to the reference method, RT-PCR. There is no doubt that these features could be further improved with industrial development for large-scale application of the methodology, and that it might even eventually outperform molecular diagnostic performance at a much lower cost while also producing faster results. Since the intensities of several conserved peptides are recorded with excellent precision and accuracy, the method presents considerable value for accurate monitoring of SARS-CoV-2 viral loads in at-risk patients, such as immunocompromised subjects with long-term treatments. Recently, whole-genome sequencing evidence was reported, demonstrating significant RNA heterogeneity in the SARS-CoV-2 virions produced during infection [8]. Some of the nucleotide changes are visible at the protein level, and would therefore be measurable by tandem mass spectrometry [9]. Specific mass spectrometry-based characterization of SARS-CoV-2 variants and investigations of this particular heterogeneity might be launched depending on the results of routine diagnostic tests. For example, this type of assay could be useful to monitor how the virus evolves over the course of long-term treatment in immunocompromised patients infected with COVID-19 in parallel to precise quantitation of viral particle loads by immuno-capture and targeted proteomics.

Since tandem mass spectrometry is already used quite extensively in analytical laboratories in clinical settings, the results presented by Renuse et al [1]. will be significant to clinical scientists. From now on, they should take the lead and request such a methodology whenever more precise quantitative results would be beneficial in their clinical practice. The same instrumental platform could be used to monitor additional markers, such as host response-specific signals, that might predict disease severity, as well as possibly identifying secondary infections linked to other pathogens.

To rival RT-PCR in terms of results and scale, some improvements must be made to mass spectrometry-based proteotyping, including: automation and acceleration of sample preparation, miniaturization of high-resolution mass spectrometers, reduced cost thanks to scaling-up and streamlining production, but also rationalization and standardization of assays. The advances afforded will be well worth the effort required. Validating this type of methodology on medical samples from large cohorts is the primary objective in the field [10]. Based on the SARS-CoV-2 detection demonstration proposed by Renuse et al [1]. and the continuous cycle of improvements to mass spectrometry instrumentation – regularly increasing sensitivity and throughput – there is no doubt that tandem mass spectrometry-based testing could be the method of choice in future pandemics.

## Conflict of interest

The author declares no conflict of interest.

## Contributors

JA conceived and wrote the entire commentary.

## Declaration of Competing Interest

None.

## References

[1] Renuse S, Vanderboom PM, Maus AD, Kemp JV, Gurtner KM, Madugundu AK (2021). A mass spectrometry-based targeted assay for detection of SARS-CoV-2 antigen from clinical specimens. EBioMedicine.

[2] Zhang L, Richards A, Barrasa MI, Hughes SH, Young RA, Jaenisch R. (2021). Reverse-transcribed SARS-CoV-2 RNA can integrate into the genome of cultured human cells and can be expressed in patient-derived tissues. Proc Natl Acad Sci U S A..

[3] Grenga L, Pible O, Armengaud J. (2019). Pathogen proteotyping: a rapidly developing application of mass spectrometry to address clinical concerns. Clin Mass Spectrom.

[4] Gouveia D, Grenga L, Gaillard JC, Gallais F, Bellanger L, Pible O (2020). Shortlisting SARS-CoV-2 peptides for targeted studies from experimental data-dependent acquisition tandem mass spectrometry data. Proteomics.

[5] Rajczewski AT, Mehta S, Nguyen DDA, Gruning B, Johnson JE, McGowan T (2021). A rigorous evaluation of optimal peptide targets for MS-based clinical diagnostics of Coronavirus Disease 2019 (COVID-19). Clin Proteomics.

[6] Gouveia D, Miotello G, Gallais F, Gaillard JC, Debroas S, Bellanger L (2020). Proteotyping SARS-CoV-2 virus from nasopharyngeal swabs: a proof-of-concept focused on a 3 min mass spectrometry window. J Proteome Res.

[7] Ihling C, Tanzler D, Hagemann S, Kehlen A, Huttelmaier S, Arlt C (2020). Mass spectrometric identification of SARS-CoV-2 proteins from gargle solution samples of COVID-19 patients. J Proteome Res.

[8] Lythgoe KA, Hall M, Ferretti L, de Cesare M, MacIntyre-Cockett G, Trebes A (2021). SARS-CoV-2 within-host diversity and transmission. Science.

[9] Gallais F, Pible O, Gaillard JC, Debroas S, Batina H, Ruat S (2021). Heterogeneity of SARS-CoV-2 virus produced in cell culture revealed by shotgun proteomics and supported by genome sequencing. Anal Bioanal Chem.

[10] Van Puyvelde B, Van Uytfanghe K, Tytgat O, Van Oudenhove L, Gabriels R, Bouwmeester R (2021). Cov-MS: a community-based template assay for mass-spectrometry-based protein detection in SARS-CoV-2 patients. JACS Au.

